# Mapping actions to promote physical activity in light of Health Promoting Schools

**DOI:** 10.1590/0034-7167-2024-0211

**Published:** 2025-11-07

**Authors:** Janiel Ferreira Felício, Isabela Natasha Pinheiro Teixeira, Victor Hugo Santos de Castro, Raquel Sampaio Florêncio, Victor José Machado de Oliveira, Laécio de Lima Araujo, Valter Cordeiro Barbosa

**Affiliations:** I Universidade Estadual do Ceará. Fortaleza, Ceará, Brazil; II Universidade Federal de Goiás. Goiânia, Goiás, Brazil

**Keywords:** Health, Child, Adolescent, School Health Services, Health Promotion., Salud, Niño, Adolescente, Servicios de Salud Escolar, Promoción de la Salud.

## Abstract

**Objectives:**

to map actions to promote physical activity and health for children and adolescents in light of the Health Promoting School model.

**Methods:**

a documentary study, mapping the proposed actions in 18 documents from the World Health Organization, the Brazilian Ministry of Health and systematic reviews on the subject. Researchers performed the convergence and summary of actions using Health Promoting School model as well as analysis of clarity and semantics of recommended actions.

**Results:**

after analyzing the 360 actions initially verified, 36 were mapped, covering governance standards (20 actions in four standards) and school context structuring (16 actions in four other standards) to be an Health Promoting School.

**Conclusions:**

this mapping is a tool to support the planning and implementation of studies and educational intervention programs that aim to develop health promotion in Brazilian schools in in light of Health Promoting School guidelines.

## RESUMEN

**Objetivos:**

mapear acciones para promover la actividad física y la salud entre niños y adolescentes a la luz del modelo de Escuela Promotora de Salud.

**Métodos:**

estudio documental, con mapeo de las acciones propuestas en 18 documentos de la Organización Mundial de la Salud, del Ministerio de Salud de Brasil y revisiones sistemáticas sobre el tema. Los investigadores realizaron la convergencia y resumen de acciones utilizando el modelo Escuela Promotora de Salud, así como el análisis de claridad y semántica de las acciones recomendadas.

**Resultados:**

luego de analizar las 360 acciones inicialmente verificadas, se mapearon 36, abarcando estándares de gobernanza (20 acciones en cuatro estándares) y estructuración del contexto escolar (16 acciones en otros cuatro estándares) para ser una Escuela Promotora de Salud.

**Conclusiones:**

este mapeo es una herramienta para subsidiar la planificación e implementación de estudios y programas de intervención educativa que busquen desarrollar la promoción de la salud en las escuelas en el contexto brasileño a la luz de las directrices Escuela Promotora de Salud.

## INTRODUCTION

Schools have historically been recognized as a fruitful space for health promotion policies, programs and actions^(1)^. Although a Health Promoting School (HPS) approach and others that seek to promote health have been in place for over 25 years, having been developed in the late 1980s with the values established in the Ottawa Charter, it was only in 2018 that the World Health Organization (WHO), in collaboration with the United Nations Educational, Scientific and Cultural Organization, announced an initiative that included a commitment to develop global standards and indicators. More recently, starting in 2021, WHO, in partnership with other institutions, has been releasing documents with recommendations to support health promotion actions in the school context^(1-18)^.

In particular, WHO detailed the categories and indicators in an implementation guide that allows “transforming every school into a HPS”. In this document, a HPS is described as one that is constantly creating and reinforcing conditions to provide a healthy setting for coexistence, learning and work. To this end, actions in eight distinct categories (called standards in the English document and translated into Portuguese) were recommended and seek to strengthen governance (management of resources and leadership) (1) government policy and resource variables (resources, investments, sectors involved in the elaboration of municipal, state and federal policy); 2) school policies and resources (action plans, objectives, priorities, goals and deadlines); 3) school governance and leadership; 4) school-community partnerships; and school context structuring (in the curriculum, in the organization of physical and social settings and healthcare services). To promote the health and development of its community, the following categories were observed: 5) school curriculum; 6) school socio-emotional setting; 7) school physical setting; and 8) healthcare services at school^(2,19)^.

These WHO-HPS categories have been fundamental for the creation of guidelines for health promotion in schools that are developed with different topics relevant to health. For instance, the action to promote physical activity in schools, developed by WHO^(3)^, is based on HPS to guide how the school context can favor conditions for an active and healthy life in children and adolescents. This ensures that physical activity promotion is permeated by relevant principles of health promotion, such as equity, social participation and empowerment/self-care^(20)^.

Although these recommendations for action have been reinforced and tested in several intervention studies over the years, the literature has highlighted the challenge of how to adapt them to everyday school life in different contexts and realities. In this regard, reviews have systematized evidence on the implementation of interventions based on WHO-HPS^(4,21-24)^ and the need to expand knowledge about possibilities of actions/strategies that can be implemented in schools and education networks that aim for an WHO-HPS in a flexible and adjustable manner to different realities^(24)^.

In Latin America, in particular, a literature review highlighted the lack of studies that could support the implementation of health promotion actions in schools^(22)^. In Brazil, a scoping review mapped more than 40 intervention studies to promote physical activity and body literacy in the country^(5)^. However, substantial actions in structuring WHO-HPS are still organized according to the dimensions represented by WHO standards on governance and school context structuring. This makes it difficult to understand how to carry out and adapt health actions in schools to ensure implementation, as proposed by WHO-HPS. Evidence points to the need for stronger alliances between health and education for the effective implementation of actions^(23,25)^, given the social determinants of health that produce severe vulnerabilities and health inequities, as in Brazil and other Latin American countries.

Therefore, it is important to map actions that have been identified as possibilities for addressing the dimensions represented by WHO-HPS standards. This will enable the synthesis of a diversity of actions that can be adapted and used according to the local context, i.e., the Brazilian reality. Furthermore, it can support reflection on elements that ratify WHO-HPS multidisciplinary and intersectoral proposal, offering health and education professionals resources for implementing the actions.

## OBJECTIVES

To map actions to promote physical activity and health for children and adolescents in light of the World Health Organization’s Health Promoting School (WHO-HES) model.

## METHODS

### Ethical aspects

Since this study used data available for public consultation and did not involve human beings, it was not forwarded for consideration by the Research Ethics Committee. However, it is important to note that the ideas of the authors who supported this research were duly cited and referenced.

### Study design

This is a documentary study that adopted mapping methodological strategies^(26)^ and followed the Preferred Reporting Items for Systematic reviews and Meta-Analyses extension for Scoping Reviews^(27)^ writing guidelines. This study design was adopted because it allows the description and summary of the state of evidence for the issue or topic under study, a stage that is therefore relevant to support implementation sciences. It is important to note that this type of study differs from reviews, since the eligible documents that served as the basis for this mapping are widely recognized and consolidated in a specific topic, and are available on the websites of research institutions or organizations that generate guidelines for the area under study. Therefore, the synthesis in mapping of actions extracted from these references allows the generation of products for evidence-based decision-making in a more targeted manner.

### Research question

The study was based on the following question: what actions to promote physical activity and health can or have been carried out/recommended so that schools meet HPS model proposed by WHO?

### Study period and sources

The study was carried out between March and December 2022, comprising the stages of extraction, deepening, reading and convergence of actions selected from the archives.

Since this is a specific proposal from WHO, the documents were identified by searching the institution’s websites and regional offices of WHO and the United Nations. The Brazilian Ministry of Health’s Primary Care Department website was also consulted to search for documents on the subject. Furthermore, systematic reviews that served as a basis for the institutional documents were also included based on the references contained in the websites. To identify the reviews published after these publications, the search equation “Health promoting school” AND Review was constructed in three databases (LILACS/via VHL, MEDLINE/via VHL and Scopus).

### Study protocol

**Figure 1:**
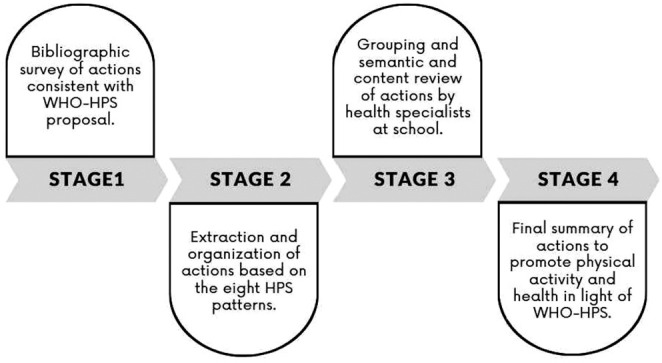
Stages of mapping physical activity and health promotion actions carried out/recommended in light of the Health Promoting School proposal

The four stages of this research were conducted to outline actions focused on the mapping issue, as shown in Figure 1.

### Stage 1 - Bibliographic survey of actions

The first stage was characterized by a bibliographic survey to support the adoption of actions that would be in line with WHO-HPS proposal. For this stage, the following criteria for inclusion of documents were considered:

1. International documents with theoretical models that underpin WHO-HPS provided fundamental conceptual support for the development of actions, in addition to being the official WHO documents for theoretical detailing of the proposal in favor of its implementation;
2. Systematic reviews that detailed interventions to promote physical activity and health in schools in different countries were included when they had WHO-HPS as their methodological basis (study selection and synthesis), either in citing the main reference on the subject^(6)^ or in WHO documents that detail WHO-HPS;
3. Documents and systematic reviews that dealt with actions to promote physical activity and health in the Brazilian reality were considered, as long as they were structured (methodological basis) with direct reference to WHO-HPS^(6)^ or organization of evidence that allowed identification of WHO-HPS standards.

Based on this process, 18 documents were analyzed, six of which were international documents based on WHO-HPS model, seven were reviews based on HPS model and five were documents and reviews based on HPS model for the Brazilian context, as shown in Chart 1 and Chart 2.

**Chart 1:** Scoping or systematic review studies that brought a Health Promoting School model, 2024 (N = 10)

Study/year	Objective	Intervention	Strategy/content covered
Brandes *et al*., 2022^(13)^ Germany	Provide an overview of school-based interventions for promoting PA, CRF and reducing SB among children aged 6-10 years, and to map these interventions to the WHO-HPS framework.	School intervention studies to promote PA, CRF and reduce SB among children aged 6 to 10 years.	Studies with information on study and intervention characteristics, effectiveness on PA, SB, CRF results and characteristics of WHO-HPS framework.
Barbosa Filho *et al*., 2021^(5)^ Brazil	Map existing evidence on interventions to promote PA and/or physical literacy components in Brazilian school-aged children and adolescents.	School-based intervention studies, with students aged 6 to 18 years, that assessed PA components, factors or attributes related to PA.	Studies that used strategies such as changes in physical education classes, extracurricular PA moments and health education, which follows WHO-HPS structure.
Dumith *et al*., 2021^(15)^ Brazil	Describe how the chapter for children and young people aged 6 to 17 years of the Physical Activity Guide for the Brazilian Population was developed and present the main recommendations for this age group.	Construction of a chapter of the guide involving the following stages: a) literature review; b) writing the preliminary version of the chapter; c) listening process with the target audience related to the chapter and experts in ​​PA promotion; d) carrying out a public consultation and; e) writing the final version of the chapter.	Topics with examples of physical activities practiced in different domains and recommendations for practice (types, intensity, frequency, duration and the ways in which it can be structured) as guidelines for young people, parents/guardians and teachers on adopting and maintaining a more physically active lifestyle, as well as suggestions for reducing time in SB.
Silva *et al*., 2021^(16)^ Brazil	Present the process of developing Brazilian recommendations for school physical education for the Brazilian population, more specifically students, teachers, parents/guardians and managers.	Synthesis of evidence, listening to the target audience and public consultation to develop recommendations.	Synthesis of 63 national and international documents with recommended strategies for school physical education, focusing on the following dimensions: policy and setting; curriculum; appropriate instruction; assessment; and strategies that interact with school physical education.
Bastos *et al*., 2020^(8)^ Brazil	Synthesize evidence on the dimensions of RE-AIM in interventions based on WHO-HPS approach in Latin America.	Studies of interventions based on WHO-HPS model carried out in Latin America involving the population aged 5 to 18 years.	Data on the implementation of the intervention based on WHO-HPS summarized in categories: 1) school curriculum; 2) changes in the social and/or physical setting of schools; and 3) actions with families and the community.
Lee *et al*., 2020^(1)^ China	Summarize studies that assessed the implementation of HPS model.	Studies assessing the implementation of WHO-HPS in schools.	Development of systems to analyze whether each school has reached the standard of an WHO-HPS.
McHugh *et al*., 2020^(10)^ United Kingdom	Investigate the effectiveness of interventions using WHO-HPS framework approach in increasing PA and improving the diet of young people aged 11-18 years.	Studies of interventions only in PA and/or nutrition.	Studies describing the implementation of PA and/or nutrition according to WHO-HPS approach.
McIsaac *et al*., 2016^(9)^ Canada	Identify research to support the adoption of a HPS approach across school systems.	Studies in nine international school systems.	The studies described policies that directed resources to schools to support the implementation of a WHO-HPS approach.
Pearson *et al*., 2015^(4)^ United Kingdom	Identify the conditions and actions that lead to the successful implementation of health promotion programs in schools.	Studies that developed health promotion programs at school.	Studies that assessed preparation for implementation, initial implementation, incorporation into routine practice, adaptation and evolution of WHO-HPS approach, compared to UK schools, allowing the identification of different contexts.
Langford *et al*., 2014^(6)^ United Kingdom	Assess the effectiveness of HPS structure in improving students’ health and well-being and their academic performance.	Cluster-randomized controlled trials in which randomization took into account the level of school, district, or other geographic area. Participants were children and youth aged 4-18 years.	Studies that address the implementation of health strategies according to WHO-HPS approach.

*PA - physical activity; WHO-HPS - World Health Organization Health Promoting School; CRF - cardiorespiratory fitness; SB - sedentary behavior; HPS - Health Promoting School.*

**Chart 2:** Institutional documents that brought recommendations on active schools and/or a Health Promoting School model, 2024 (N = 8)

Author/year	Country	Publication type and main content
Active Schools, 2022^(7)^	United States of America	Literature review - provide school communities with a guide with strategies for implementing active schools.
Boronyai *et al*., 2022^(12)^	Luxembourg	Literature review - compile the dimensions and layers that represent the functions and setting of a school from the point of view of promoting PA.
WHO, 2021^(3)^	Switzerland	Literature review - provide tools to support countries seeking to develop and implement effective policy actions to increase PA practice in schools.
WHO, 2021^(11)^	Switzerland	Literature review - assist national, state and local governments in developing, planning, financing and monitoring sustainable approaches to health promotion in schools that address the health and well-being of students, parents, caregivers, school staff and local communities.
WHO, 2021^(2)^	Switzerland	Literature review - support the implementation of HPS through government departments and ministries, school staff, civil society entities and partners.
Brasil, 2021^(17)^	Brazil	Literature review - bring the first recommendations and information from the Ministry of Health on PA so that the population can have an active life, promoting health and improving quality of life.
Bada *et al*., 2019^(14)^	Greece, France and Portugal	Literature review - synthesize European standards and indicators for HPS according to school stakeholders’ perceptions.
Brasil, 2016^(18)^	Brazil	Literature review - contribute to a reflection on how to make schools a space conducive to human development, improving understanding of the role of physical and sports activities in this context and, consequently, educational policies in the area.

*WHO - World Health Organization; HPS - Health Promoting School; PA - physical activity.*

### Stage 2 - Extraction of actions according to the World Health Organization’s Health Promoting School model

After the bibliographic survey, stage 2 began with the reading of the materials in full. The qualitative information reported was recorded and organized using the content analysis technique, structuring the data for better interpretation. No qualitative tool was used. Categorical analysis classifies the constituent elements of a set by differentiation, regrouping them according to common characteristics^(28)^. In this way, a pre-analysis of collected material was carried out in order to organize those that had a pattern to be analyzed. After this stage, significant excerpts were coded, and, finally, they were categorized according to WHO-HPS dimension, making the semantic, phrasal, expression convergence and the frequency with which they appeared (see supplementary material), followed by the extraction of actions that addressed aspects related to the eight WHO-HPS standards. All excerpts that made clear the action (verbs such as build, participate, execute, among others) or a construct of physical activity and health that could be promoted and that was articulated with the WHO-HPS eight standards were considered: 1) variables of government policies and resources (resources, investments, sectors involved in the elaboration of municipal, state and federal policy); 2) school policies and resources (action plans, objectives, priorities, goals and deadlines); 3) school governance and leadership; 4) school-community partnerships; 5) school curriculum; 6) school socio-emotional setting; 7) school physical setting; and 8) healthcare services at school^(2,19)^.

All actions were extracted and organized into charts according to standards. Documents and reviews in the original language and translated into the final version were included. This process was carried out by a list of four researchers, and reviewed by another author. They were trained based on the action selection criteria and WHO-HPS standards to avoid selection and analysis errors. Consensus meetings were held weekly to monitor the process of extracting the actions. After that, the meetings were aligned to review the content and actions that dialogued with WHO-HPS model. At the end of this stage, the general list of all physical activity and health promotion actions was generated (see supplementary material 1, “extraction” tab).

### Stage 3 - Part 1: Grouping and semantic review of action convergences

Stage 3 consisted of grouping and organizing actions that presented a similar operational direction (e.g., similar actions and were carried out in the classroom) for the WHO-HPS eight standards. This process was carried out because it allowed a thematic synthesis that considered the multidimensionality and complexity of extracted content. The dynamic and deductive-inductive process (i.e., groupings were created and discussed in groups until theoretical saturation was achieved) represents an essential procedure for synthesizing evidence on complex issues^(29)^.

The process of organizing and categorizing the content was carried out during extraction by one of the four authors, and was validated in consensus meetings among the other authors to ensure that the topics found were correctly grouped and categorized until theoretical saturation of the topics was achieved.

This construction was primarily based on reflections based on WHO-HPS model. During the process, aspects related to the Brazilian context were also presented, with the aim of creating a facilitating mapping for the insertion of this recommendation into practice. Therefore, the rewriting of actions occurred through their refinement, especially through textual review among researchers.

After the grouping process, the same reviewers summarized WHO-HPS recommendations aimed at promoting physical activity and health in schools. The final version of actions was structured considering the following elements: the action performed (represented by the verb in the infinitive); the recommended strategy (focused on HPS principle or its contribution to health); the context (target audience); and HPS dimensions involved (governance, school resources, curriculum, community, healthcare professionals, teachers, students). Thus, this stage was concluded with the preliminary version of actions (see supplementary material 1, “convergences” tab).

### Stage 3 - Part 2: semantic and content review of actions

In the third stage, actions were reviewed semantically and in content by experts who research health in schools. Experts were identified because they were authors of several scientific studies on physical activity and health in schools, based on the list of references of review articles included in this study. Then, snowball sampling was also applied to indicate other experts for this stage. To this end, ten expert evaluators in the area were invited to participate in the final review of the text of actions. Of these, only five returned with suggestions for improving the writing of actions, including reviewing the chosen verbs, seeking their standardization and thus improving their understanding.

### Stage 4 - Final summary of actions

Finally, the process of adaptation to criticisms and suggestions was carried out to create the final version of the mapping. Due to the volume of suggestions, new consensus meetings were necessary for correction and adaptation. With this, the final version of actions to promote physical activity and health in light of WHO-HPS was established (see supplementary material 2).

## RESULTS

**Figure 2:**
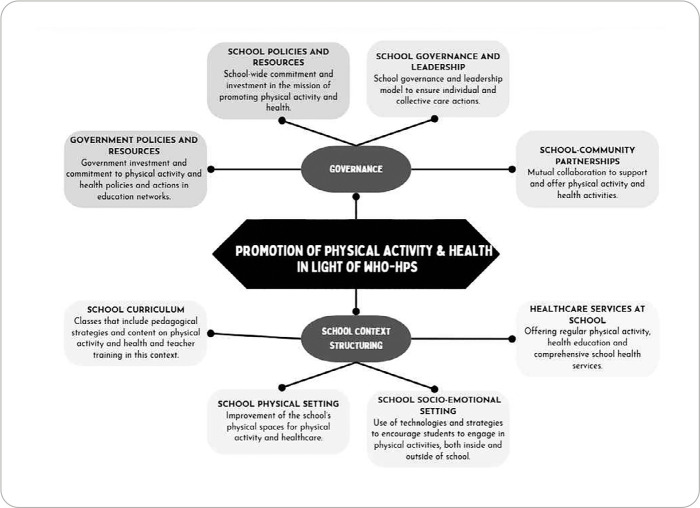
Actions to promote physical activity and health in light of a Health Promoting School

In the extraction process (first stage), the 18 documents assessed allowed the identification of 360 distinct actions, which were organized according to the WHO-HPS eight standards (see supplementary material 1). The convergence stage allowed the alignment, reorganization and grouping of actions that had similar content. After several consensus and theoretical saturation meetings, these actions were grouped into 36 actions, covering the WHO-HPS proposal eight standards (Figure 2). The finalized actions, after the semantic and content analysis process by experts, were compiled in a chart (see supplementary material 2).

*WHO-HPS - World Health Organization’s Health Promoting School.*

In total, 20 actions covered the four standards on school governance: government policies and resources (eight actions; 22.22%); school policies and resources (five actions; 13.9%); school governance and leadership (three actions; 8.33%); school-community partnerships (four actions; 11.11%).

A total of 16 actions were also mapped, covering the four standards on structuring the school context: school curriculum (seven actions; 19.44%); school socio-emotional setting (three actions; 8.33%); school physical setting (three actions; 8.33%); and healthcare services at school (three actions; 8.33%).

## DISCUSSION

This documentary research presented a mapping of actions that can be a tool to support the planning and implementation of studies and intervention programs that aim to develop the promotion of physical activity and health in schools in the Brazilian context and that are based on WHO-HPS recommendations.

The strategy map has a greater number of actions focused on governance. In particular, actions on government and school policies and resources are directed towards governance that involves the availability of financial and material resources to be allocated to schools to enable an educational network that is committed to promoting health. This highlights that the agenda for promoting physical activity and health in schools involves ensuring resources, governance and political commitment to building settings with favorable conditions and opportunities for physical activity in students’ and school community’s lives^(2-18)^.

Seven actions were mapped regarding school leadership and management, with emphasis on including the community in actions to promote physical activity and health at school. This is a challenge, since alternatives will have to be thought of and worked on to involve individuals on an ongoing basis. In order for health promotion actions to have greater added value in participants’ lives, they need to be maintained for a long time. These actions have multiple influences on the lives of people involved in this process, since, in order for healthy conditions to last, a support network that coincides with these perspectives is needed. In other words, a HPS proposal construction involves ongoing aspects of health promotion^(30,31)^.

The 16 actions for structuring the school context had a greater concentration of actions involving curricular components (n=7). This denotes part of the challenges encountered in the proposal, given that it centralizes in curriculum responsibilities related to different purposes, such as academic performance. A study indicates that several factors contribute to academic performance, with emphasis on the curriculum in settings with intersectoral collaborative learning^(32)^.

Most of the actions observed involved the development of physical activities in schools, addressing topics with dimensions of healthy practices with a focus on both the individual and collective levels^(12,30)^. This is in line with HPS model, since the dimensions must involve health promotion practices that go beyond physical activity, including an integral perspective and a holistic view of human development. In other words, in addition to a behavioral aspect, it is necessary to consider the social, cultural and emotional aspects that influence and need to be addressed in school health promotion policies^(9,33)^. Hence, we can say that the promotion of physical activity in school settings tend to gain a broader focus when it is treated as a pedagogical issue linked to health education^(34,35)^.

Actions still arise, most of the time, from national physical activity plans^(12,13,17,31)^. In view of this, many focus on physical activity in school settings, which is seen as one of the priority strategies in the documents. The importance of this element in promoting school health is reinforced, but attention is drawn to the importance of expanding actions that involve aspects related to school members’ mental health. Studies point to the need to address this issue, since it has a direct impact on the physical health of students^(36-39)^.

There is a need for changes to the curriculum. However, this strategy is hindered by a system in which national guidelines are based on biological divisions, ignoring the intersectoral nature of the topics. Therefore, the curricula reproduce technical biases and HPS strategy assumes interrelated prevention and promotion practices.

### Study limitations

Some limitations of this study need to be highlighted. The search was restricted to documents published in English and Portuguese. This is also because HPS model is not yet widely disseminated, with the studies found coming from more comprehensive school health actions and with specific topics as previously mentioned. Furthermore, the convergence of the 36 actions represents a strategy to dimensionally several actions. However, details of each possible action end up being hidden when grouping them. Therefore, the analysis of convergence of the 36 actions by readers should be accompanied by the initial list of actions (see supplementary material 1) so that it is possible to analyze and adapt the details of actions to each reality and possibility.

### Contributions to nursing, health, or public policy

The documents with WHO-HPS^(2,19)^ standards and indicators direct their recommendations to the groups of school stakeholders who can collaborate to promote health in the aforementioned context, including researchers on school health, policy and program makers, school administrators, representatives of school councils and committees, teachers, healthcare professionals, students, and family members. For the Brazilian context, it is expected that the mapping of actions presented here can support these stakeholders in their actions, with potential implications for pedagogical and research practices, namely:

- Governance actions (standards 1 to 4) can be considered by health managers and professionals (in dialogue with education managers and professionals) to align public policies and municipal, state and federal programs in the development and management of school health programs. Education networks can include in their public health policy implementation plans the increase in actions based on this strategy map adapted to different realities and that nurture a system with greater potential for implementation, effectiveness and sustainability;
- School managers, coordinators and teachers can also include the implementation of WHO-HPS actions in school routine, including in the Pedagogical Political Project to organize the policy, resources, methodologies, and social involvement and engagement in the promotion of physical activity and health at school.
- Teachers of both conventional curricular components (physical education, mathematics, science, arts, among others) and those who work in schools with innovative curricular proposals (with elective curricular components or a diversified base) can consider the curricular actions proposed here for the formulation of projects and pedagogical actions as a way of guaranteeing an intersectoral and interdisciplinary approach to learning in health that has real meaning in people’s lives;
- The entire school community can support the implementation of actions to develop the physical and socio-emotional setting that promote healthcare and are appropriate to different realities and contexts. In this sense, the community must be encouraged by participatory management in the development of a collective policy.

This study provides the basis for the creation of scientifically refined proposals for actions and strategies, which include the social actors involved in this process. Future research may include the implementation of these actions and strategies in different local and regional contexts of the country, as it will provide a more comprehensive assessment. In this sense, an assessment of the implementation of actions and strategies of this nature is relevant, considering the economic, social and cultural aspects of the countries.

## CONCLUSIONS

The mapping of actions based on WHO-HPS model allowed us to identify the convergence of 36 actions that can serve as a basis for the development of governance and organization of Brazilian schools, favoring the promotion of physical activity and health in light of international models and standards for health promotion in schools. Studies and interventions can consider and rely on this mapping to promote the implementation of public policies and programs for health education and health promotion in Brazil and other similar contexts.

## Data Availability

The research data are available in a repository: https://doi.org/10.48331/scielodata.8WDPXC.
