# Supplementary material for: Mapping actions to promote physical activity in light of Health Promoting Schools
Source: Rev Bras Enferm. 2025 Nov 7;78(4):e20240211. doi: 10.1590/0034-7167-2024-0211 (PMC12594482; doi:10.1590/0034-7167-2024-0211)
Supplement: Translation

# Translation: Mapeando ações para a promoção da atividade física à luz da Escola Promotora de Saúde

**DOI:** 10.1590/0034-7167-2024-0211pt

**Authors:** Janiel Ferreira Felício, Isabela Natasha Pinheiro Teixeira, Victor Hugo Santos de Castro, Raquel Sampaio Florêncio, Victor José Machado de Oliveira, Laécio de Lima Araujo, Valter Cordeiro Barbosa

**Affiliations:** I Universidade Estadual do Ceará. Fortaleza, Ceará, Brasil; II Universidade Federal de Goiás. Goiânia, Goiás, Brasil

**Keywords:** Saúde, Criança, Adolescente, Serviços de Saúde Escolar, Promoção da Saúde.

## Abstract

**Objetivos:**

mapear ações de promoção da atividade física e saúde de crianças e adolescentes à luz do modelo Escola Promotora da Saúde.

**Métodos:**

estudo documental, com mapeamento das ações propostas em 18 documentos da Organização Mundial da Saúde, Ministério da Saúde brasileiro e revisões sistemáticas sobre o tema. Pesquisadores realizaram a convergência e sumarização destas pelo modelo Escola Promotora da Saúde, como a análise de clareza e semântica das ações recomendadas.

**Resultados:**

após análise das 360 ações inicialmente verificadas, 36 foram mapeadas, contemplando os padrões de governança (20 ações em quatro padrões) e estruturação do contexto escolar (16 ações em outros quatro padrões) para ser uma Escola Promotora da Saúde.

**Conclusões:**

este mapeamento é uma ferramenta para apoiar o planejamento e implementação de estudos e programas de intervenção educativa que almejam desenvolver a promoção da saúde na escola no contexto brasileiro à luz das diretrizes da Escola Promotora da Saúde.

## INTRODUÇÃO

As escolas são historicamente reconhecidas como um espaço profícuo para políticas, programas e ações de promoção da saúde^(1)^. Embora a abordagem Escola Promotora de Saúde (EPS) e outras que buscam a promoção da saúde sejam realizadas há mais de 25 anos, sendo desenvolvida no final da década de 1980 com os valores estabelecidos na Carta de Ottawa, somente em 2018, a Organização Mundial da Saúde (OMS), em colaboração com a Organização das Nações Unidas para a Educação, Ciência e Cultura, anunciou uma iniciativa que incluía um compromisso de desenvolver padrões e indicadores globais. Mais recentemente, a partir de 2021, a OMS, em parceria com outras instituições, tem lançado documentos com recomendações para apoiar ações de promoção da saúde no contexto escolar^(1-18)^.

Em particular, a OMS detalhou as categorias e indicadores em um guia de implementação que permite “transformar toda escola em uma EPS”. Nesse documento, uma EPS é descrita como aquela que está constantemente criando e reforçando condições para propiciar um entorno saudável para o convívio, aprendizagem e trabalho. Para isso, ações em oito categorias distintas (chamadas de padrões no documento em inglês e traduzido ao português) foram recomendadas e buscam fortalecer a governança (gestão de recursos e liderança) (1) variáveis de políticas e recursos governamentais (recursos, investimentos, setores envolvidos na elaboração da política municipal, estadual e federal); 2) políticas e recursos da escola (planos de ação, objetivos, prioridades, metas e prazos); 3) governança e liderança da escola; 4) parcerias da escola com a comunidade) e a estruturação do contexto escolar (no currículo, na organização dos ambientes físicos e sociais e dos serviços de saúde). Para favorecer a saúde e o desenvolvimento da sua comunidade, foram observados nas categorias: 5) currículo da escola; 6) ambiente socioemocional da escola; 7) ambiente físico da escola; e 8) serviços de saúde na escola^(2,19)^.

Essas categorias da EPS-OMS têm sido basilares para a criação de orientações de promoção da saúde na escola que sejam desenvolvidas com diferentes temas relevantes à saúde. Por exemplo, a ação de promoção da atividade física na escola, elaborada pela OMS^(3)^, fundamenta-se na EPS para orientar como o contexto escolar pode favorecer condições para uma vida ativa e saudável em crianças e adolescentes. Isso garante que a promoção da atividade física seja permeada por princípios relevantes da promoção da saúde, como equidade, participação social e empoderamento/autocuidado^(20)^.

Embora essas recomendações de ações sejam reforçadas e testadas em diversos estudos de intervenção ao longo de anos, a literatura tem destacado o desafio de como adequá-las ao cotidiano escolar em diferentes contextos e realidades. Nesse sentido, revisões têm sistematizado evidências sobre a implementação de intervenções baseadas na EPS-OMS^(4,21-24)^ e a necessidade de ampliar o conhecimento sobre as possibilidades de ações/estratégias que possam ser implementadas em escolas e redes de ensino que almejam uma EPS-OMS, de modo flexível e ajustável às diversas realidades^(24)^.

Na América Latina, em particular, revisão bibliográfica apontou a carência de estudos que possam apoiar a implementação de ações de promoção da saúde na escola^(22)^. No Brasil, revisão de escopo mapeou mais de 40 estudos de intervenção de promoção da atividade física e do letramento corporal no país^(5)^. No entanto, as ações substanciais na estruturação das EPS-OMS ainda são organizadas conforme as dimensões representadas pelos padrões da OMS sobre governança e estruturação do contexto escolar. Isso dificulta sua compreensão de como realizar e adequar as ações de saúde na escola para garantir a implementação, conforme a proposta construída pela EPS-OMS. As evidências apontam para necessidade de alianças mais fortes entre a saúde e educação para implementação efetiva das ações^(23,25)^, diante de determinantes sociais da saúde que produzem severas vulnerabilidades e iniquidades em saúde, como no Brasil e outros países da América Latina.

Dessa maneira, torna-se relevante a realização do mapeamento de ações que têm sido evidenciadas como possibilidades de contemplar as dimensões representadas pelos padrões da EPS-OMS. Isso possibilitará a síntese de uma diversidade de ações que possam ser adequadas e utilizadas de acordo com o contexto local, ou seja, a realidade brasileira. Ademais, poderá dar subsídio à reflexão acerca de elementos que ratificam a proposta multiprofissional e intersetorial da EPS-OMS, oferecendo aos profissionais de saúde e de educação recursos para implementação das ações.

## OBJETIVOS

Mapear as ações de promoção da atividade física e saúde de crianças e adolescentes à luz do modelo Escola Promotora da Saúde da Organização Mundial da Saúde (EPS-OMS).

## MÉTODOS

### Aspectos éticos

Por se tratar de estudo que utilizou dados disponíveis para consulta pública e não envolver seres humanos, não foi encaminhado para apreciação pelo Comitê de Ética em Pesquisa. No entanto, destaca-se que as ideias dos autores que subsidiaram essa pesquisa foram devidamente citadas e referenciadas.

### Desenho do estudo

Trata-se de estudo documental que adotou estratégias metodológicas de mapeamento^(26)^ e seguiu as diretrizes de escrita da *Preferred Reporting Items for Systematic reviews and Meta-Analyses extension for Scoping Reviews^(^* ^27)^. Este desenho de estudo foi adotado por permitir a descrição e sumarização do estado de evidência para questão ou tópico em estudo, etapa, portanto, relevante para fundamentar as ciências da implementação. Destaca-se que esse tipo de estudo se diferencia de revisões, pois os documentos elegíveis e que serviram de base para esse mapeamento são amplamente reconhecidos e consolidados em uma temática específica, e estão disponíveis em sites de instituições de pesquisa ou de organizações que geram diretrizes para a área em estudo. Portanto, a síntese em mapeamento das ações extraídas desses referenciais possibilita a geração de produtos para a tomada de decisão baseada em evidências de forma mais direcionada.

### Questão de pesquisa

O estudo foi baseado na seguinte pergunta: quais ações de promoção da atividade física e saúde podem ou têm sido realizadas/recomendadas para que escolas atendam ao modelo EPS proposto pela OMS?

### Período e fontes do estudo

O estudo foi realizado entre março e dezembro de 2022, compreendendo as etapas de extração, aprofundamento, leitura e convergências das ações selecionadas dos arquivos.

Por se tratar de uma proposta específica da OMS, os documentos foram identificados por meio da busca nos sítios eletrônicos da instituição e de escritórios regionais da OMS e da Organização das Nações Unidas. O portal da Secretaria de Atenção Primária do Ministério da Saúde brasileiro também foi consultado para buscar documentos sobre o tema. Ademais, revisões sistemáticas que serviram de base para os documentos institucionais também foram incluídas a partir da consulta às referências contidas nos sítios eletrônicos. Para identificar as revisões publicadas em períodos posteriores a essas publicações, foi construída a equação de busca “*Health promoting school*” *AND Review* em três bases de dados (LILACS/via BVS, MEDLINE/via BVS e Scopus).

### Protocolo do estudo

As quatro etapas desta pesquisa foram conduzidas para esquematizar ações focadas na questão do mapeamento, conforme a Figura 1.

**Figura 1:** Etapas do mapeamento das ações de promoção da atividade física e saúde realizadas/recomendadas à luz da proposta da Escola Promotora de Saúde

### Etapa 1 - Levantamento bibliográfico das ações

A primeira etapa foi caracterizada pelo levantamento bibliográfico para amparar a adoção de ações que dialogassem com a proposta da EPS-OMS. Para esta etapa, os seguintes critérios para inclusão dos documentos foram considerados:

1. Documentos internacionais com modelos teóricos que fundamentam a EPS-OMS permitiram suporte conceitual fundamental para o desenvolvimento das ações, além de serem os documentos oficiais da OMS para detalhamento teórico da proposta em prol de sua implementação;
2. Revisões sistemáticas que detalharam intervenções de promoção da atividade física e saúde em escolas de diferentes países foram incluídas quando tinham como base metodológica (seleção e síntese dos estudos) a EPS-OMS, seja na citação da principal referência sobre o tema^(6)^ ou de documentos da OMS que detalham a EPS-OMS;
3. Documentos e revisões sistemáticas que tratavam sobre ações de promoção da atividade física e saúde na realidade brasileira foram considerados, desde que fossem estruturadas (base metodológica) a referência direta da EPS-OMS^(6)^ ou a organização das evidências que permitissem identificar os padrões contemplados na EPS-OMS.

Com base nesse processo, 18 documentos foram analisados, sendo que seis eram documentos internacionais fundamentados no modelo EPS-OMS, sete eram revisões fundamentadas no modelo EPS e cinco eram documentos e revisões fundamentadas no modelo EPS para o contexto brasileiro, conforme Quadro 1 e Quadro 2.

**Quadro 1:** Estudos de revisão de escopo ou sistemática que trouxeram o modelo Escola Promotora de Saúde, 2024 (N = 10)

Estudo/ano	Objetivo	Intervenção	Estratégia/conteúdo abordado
Brandes *et al*., 2022^(13)^ Alemanha	Fornecer uma visão geral das intervenções escolares para a promoção da AF, ACR e a redução de CS entre crianças de 6 a 10 anos de idade, e mapear essas intervenções para a estrutura de EPS-OMS.	Estudos de intervenção escolar para a promoção da AF, ACR e a redução de CS entre crianças de 6 a 10 anos de idade.	Estudos com informações sobre características do estudo e da intervenção, eficácia nos resultados de AF, CS, ACR e características da estrutura EPS-OMS.
Barbosa Filho *et al*., 2021^(5)^ Brasil	Mapear as evidências existentes sobre intervenções para promover AF e/ou componentes de alfabetização física em crianças e adolescentes brasileiros em idade escolar.	Estudos de intervenção baseados em escolas, com alunos de 6 a 18 anos, que avaliaram componentes de AF, fatores ou atributos relacionados à AF.	Estudos que utilizaram estratégias como mudanças nas aulas de educação física, momentos extracurriculares de AF e educação em saúde, que segue a estrutura da EPS-OMS.
Dumith *et al*., 2021^(15)^ Brasil	Descrever como foi desenvolvido o capítulo para crianças e jovens de 6 a 17 anos do Guia de Atividade Física para População Brasileira e apresentar as principais recomendações para essa faixa etária.	Construção de um capítulo do guia envolvendo as seguintes etapas: a) revisão de literatura; b) redação da versão preliminar do capítulo; c) processo de escuta com o público-alvo referente ao capítulo e especialistas da área de promoção da AF; d) realização de consulta pública e; e) redação da versão final do capítulo.	Tópicos com exemplos de atividades físicas praticadas em diferentes domínios e recomendações para a prática (tipos, intensidade, frequência, duração e as formas que a mesma pode ser estruturada) como orientações para jovens, pais/responsáveis e professores sobre a adoção e manutenção de um estilo de vida mais ativo fisicamente, bem como sugestões para reduzir o tempo em CS.
Silva *et al*., 2021^(16)^ Brasil	Apresentar o processo de elaboração das recomendações brasileiras de educação física escolar para a população brasileira, mais especificamente os estudantes, professores, pais/responsáveis e gestores.	Síntese de evidências, escutas ao público-alvo e consulta pública para a construção das recomendações.	Síntese de 63 documentos nacionais e internacionais com estratégias recomendadas para a educação física escolar, com foco nas seguintes dimensões: política e ambiente; currículo; instrução apropriada; avaliação; e estratégias que interagem com a educação física escolar.
Bastos *et al*., 2020^(8)^ Brasil	Sintetizar evidências sobre as dimensões do RE-AIM em intervenções baseadas na abordagem EPS-OMS na América Latina.	Estudos de intervenções baseadas no modelo EPS-OMS realizadas na América Latina envolvendo a população de 5 a 18 anos.	Dados sobre a implementação da intervenção baseada na EPS-OMS resumidos em categorias: 1) currículo escolar; 2) mudanças no ambiente social e/ou físico das escolas; e 3) ações com as famílias e a comunidade.
Lee *et al*., 2020^(1)^ China	Sumarizar estudos que avaliaram a implementação do modelo de EPS.	Estudos que avaliam a implementação da EPS-OMS nas escolas.	Desenvolvimento de sistemas para analisar se cada escola atingiu o padrão de uma EPS-OMS.
McHugh *et al*., 2020^(10)^ Reino Unido	Investigar a eficácia das intervenções usando a abordagem da estrutura de EPS-OMS no aumento da AF e na melhoria da dieta de jovens de 11 a 18 anos.	Estudos de intervenções somente de AF e/ou nutrição.	Estudos que descrevessem a implementação da AF e/ou nutrição conforme abordagem EPS-OMS.
McIsaac *et al*., 2016^(9)^ Canadá	Identificar pesquisas para dar suporte à adoção de uma abordagem EPS em todos os sistemas escolares.	Estudos em nove sistemas escolares internacionais.	Os estudos descreveram políticas que direcionavam recursos para as escolas dando suporte à implementação de uma abordagem EPS-OMS.
Pearson *et al*., 2015^(4)^ Reino Unido	Identificar as condições e ações que levam à implementação bem-sucedida de programas de promoção da saúde em escolas.	Estudos que desenvolveram programas de promoção da saúde na escola.	Estudos que avaliaram a preparação para implementação, implementação inicial, incorporação na prática de rotina, adaptação e evolução da abordagem EPS-OMS, comparados às escolas do Reino Unido, permitindo identificar os diferentes contextos.
Langford *et al*., 2014^(6)^ Reino Unido	Avaliar a eficácia da estrutura das EPS na melhoria da saúde e bem-estar dos alunos e seu desempenho acadêmico.	Ensaios controlados randomizados por *cluster* em que a randomização levou em consideração o nível da escola, distrito ou outra área geográfica. Participantes foram crianças e jovens de 4 a 18 anos.	Estudos que abordassem a implementação de estratégias de saúde conforme abordagem EPS-OMS.

*AF - atividade física; EPS-OMS - Escola Promotora de Saúde da Organização Mundial da Saúde; ACR - aptidão cardiorrespiratória; CS - comportamento sedentário; EPS - Escola Promotora de Saúde.*

**Quadro 2:** Documentos institucionais que trouxeram recomendações sobre escolas ativas e/ou o modelo Escola Promotora de Saúde, 2024 (N = 8)

Autor/ano	País	Tipo de publicação e conteúdo principal
*Active Schools*, 2022^(7)^	Estados Unidos da América	Revisão de literatura - fornecer às comunidades escolares um guia com estratégias para implementação de escolas ativas.
Boronyai *et al*., 2022^(12)^	Luxemburgo	Revisão de literatura - Compilar as dimensões e camadas que representam as funções e o ambiente de uma escola do ponto de vista da promoção da AF.
WHO, 2021^(3)^	Suíça	Revisão de literatura - trazer ferramentas para apoiar os países que buscam desenvolver e implementar ações políticas eficazes para aumentar a prática de AF nas escolas.
WHO, 2021^(11)^	Suíça	Revisão de literatura - auxiliar os governos nacional, estadual e local no desenvolvimento, planejamento, financiamento e monitoramento de abordagens sustentadas para a promoção de saúde nas escolas que busque a saúde e o bem-estar de estudantes, pais, cuidadores, funcionários da escola e comunidades locais.
WHO, 2021^(2)^	Suíça	Revisão de literatura - apoiar a implementação de EPS por meio das secretarias e ministérios governamentais, equipe escolar, entidades da sociedade civil e parceiros.
Brasil, 2021^(17)^	Brasil	Revisão de literatura - trazer as primeiras recomendações e informações do Ministério da Saúde sobre AF para que a população tenha uma vida ativa, promovendo a saúde e melhoria da qualidade de vida.
Bada *et al*., 2019^(14)^	Grécia, França e Portugal	Revisão de literatura - sintetizar os padrões e indicadores europeus para EPS conforme as percepções dos atores da escola.
Brasil, 2016^(18)^	Brasil	Revisão de literatura - colaborar para uma reflexão sobre como fazer da escola um espaço propício ao desenvolvimento humano, melhorando a compreensão sobre o papel das atividades físicas e esportivas nesse contexto e, consequentemente, as políticas educacionais na área.

*WHO - World Health Organization; EPS - Escola Promotora de Saúde; AF - atividade física.*

### Etapa 2 - Extração das ações conforme o modelo Escola Promotora de Saúde da Organização Mundial da Saúde

Após o levantamento bibliográfico, a etapa 2 foi iniciada com a leitura dos materiais na íntegra. As informações qualitativas relatadas foram registradas e organizadas por meio da técnica da análise de conteúdo, estruturando os dados para melhor interpretação. Não foi usada nenhuma ferramenta qualitativa. A análise categorial classifica os elementos constitutivos de um conjunto por diferenciação, reagrupando-os de acordo com as características em comum^(28)^. Dessa maneira, realizou-se uma pré-análise do material coletado com o intuito de organizar aqueles que tinham algum padrão a ser analisado. Após essa etapa, codificaram-se os trechos significativos, e, por fim, foi feita sua categorização de acordo com a dimensão da EPS-OMS, fazendo a convergência semântica, frasal, de expressões e ad frequência com que apareciam (ver material suplementar), seguindo-se com a extração de ações que abordavam aspectos relacionados aos oito padrões da EPS-OMS. Foram considerados todos os trechos que deixavam clara a ação (verbos como construir, participar, executar, entre outros) ou um construto da atividade física e saúde que pudesse ser promovido e que estivesse articulado com os oito padrões da EPS-OMS: 1) variáveis de políticas e recursos governamentais (recursos, investimentos, setores envolvidos na elaboração da política municipal, estadual e federal); 2) políticas e recursos da escola (planos de ação, objetivos, prioridades, metas e prazos); 3) governança e liderança da escola; 4) parcerias da escola com a comunidade; 5) currículo da escola; 6) ambiente socioemocional da escola; 7) ambiente físico da escola; e 8) serviços de saúde na escola^(2,19)^.

Todas as ações foram extraídas e organizadas em quadros, de acordo com os padrões. Documentos e revisões na língua original e traduzidos na versão final foram contemplados. Esse processo foi realizado por uma lista de quatro pesquisadores, sendo revisado por outro autor. Esses foram treinados com base nos critérios de seleção das ações e nos padrões da EPS-OMS, para evitar erros de seleção e análise. Reuniões de consenso aconteceram semanalmente para acompanhamento processual da extração das ações. Após isso, as reuniões foram alinhadas para revisão dos conteúdos e das ações que dialogavam com modelo EPS-OMS. Ao final dessa etapa, a lista geral de todas as ações de promoção da atividade física e saúde foi gerada (ver material suplementar 1, aba “extração”).

### Etapa 3 - Parte 1: agrupamento e revisão semântica das convergências de ações

A etapa 3 consistiu em agrupar e organizar as ações que apresentaram um direcionamento operacional semelhante (por exemplo, ações similares e serem feitas em sala de aula) para os oito padrões da EPS-OMS. Esse processo foi realizado por permitir uma síntese temática que considerasse a multidimensionalidade e complexidade do conteúdo extraído. O processo dinâmico e dedutivo-indutivo (ou seja, que os agrupamentos eram criados e discutidos em grupo até uma saturação teórica) representa um procedimento essencial para sintetizar evidências sobre questões complexas^(29)^.

O processo de organização e categorização do conteúdo foi realizado durante a extração por um dos quatro autores, sendo validado em reuniões de consenso entre os demais autores para garantir que os tópicos encontrados fossem corretamente agrupados e categorizados até a saturação teórica dos temas.

Essa construção se deu, primariamente, por meio de reflexões embasadas no modelo EPS-OMS. No decorrer do processo, aspectos relacionados ao contexto brasileiro também foram apresentados, com o intuito de criar um mapeamento facilitador para a inserção dessa recomendação na prática. Portanto, a reescrita das ações ocorreu por meio do seu refinamento, sobretudo da revisão textual entre os pesquisadores.

Após o processo de agrupamento, os mesmos revisores resumiram as recomendações relacionadas à EPS-OMS direcionadas para promoção da atividade física e saúde na escola. A versão final das ações foi estruturada considerando os seguintes elementos: a ação realizada (representada pelo verbo no infinitivo); a estratégia recomendada (voltada ao princípio da EPS ou sua contribuição para a saúde); o contexto (público-alvo); e as dimensões da EPS envolvidas (governança, recursos da escola, currículo, comunidade, profissionais de saúde, professores, alunos). Com isso, essa etapa foi concluída com a versão preliminar das ações (ver material suplementar 1, aba “convergências”).

### Etapa 3 - Parte 2: revisão semântica e de conteúdo das ações

Ainda na terceira etapa, houve a revisão semântica e de conteúdo das ações por especialistas que pesquisam sobre saúde na escola. Os especialistas foram identificados por serem autores de diversos estudos científicos sobre o tema de atividade física e saúde na escola, a partir da lista de referências dos artigos de revisão contemplados neste estudo. Em seguida, a técnica de bola de neve também foi aplicada para indicação de outros especialistas para esta etapa. Para tanto, dez avaliadores especialistas da área foram convidados a participar da revisão final do texto das ações. Desses, apenas cinco retornaram, com sugestões para melhoria da escrita das ações, incluindo a revisão dos verbos escolhidos, buscando sua padronização e aperfeiçoando, assim, sua compreensão.

### Etapa 4 - Síntese final de ações

Por fim, deu-se o processo de adaptação às críticas e sugestões para a criação da versão final do mapeamento. Devido ao volume de sugestões, novas reuniões de consenso foram necessárias para correção e adequação. Com isso, foi estabelecida a versão final das ações de promoção da atividade física e saúde à luz da EPS-OMS (ver material suplementar 2).

## RESULTADOS

No processo de extração (primeira etapa), os 18 documentos avaliados permitiram identificar 360 ações distintas, as quais foram organizadas conforme os oito padrões da EPS-OMS (ver material suplementar 1). A etapa de convergências permitiu um alinhamento, reorganização e agrupamento das ações que tinham conteúdos semelhantes. Após diversas reuniões de consenso e saturação teórica, essas ações foram agrupadas em 36 ações, contemplando os oito padrões da proposta EPS-OMS (Figura 2). As ações finalizadas, após o processo de análise semântica e de conteúdo pelos especialistas, foram compiladas em um quadro (ver material suplementar 2).

**Figura 2:** Ações de promoção da atividade física e saúde à luz da Escola Promotora de Saúde

*EPS-OMS - Escola Promotora de Saúde da Organização Mundial da Saúde.*

Ao todo, 20 ações contemplavam os quatro padrões sobre governança da escola: políticas e recursos governamentais (oito ações; 22,22%); políticas e recursos da escola (cinco ações; 13,9%); governança e liderança da escola (três ações; 8,33%); parcerias entre escola e comunidade (quatro ações; 11,11%).

Um total de 16 ações também foram mapeadas, contemplando os quatro padrões sobre a estruturação do contexto escolar: currículo escolar (sete ações; 19,44%); ambiente socioemocional da escola (três ações; 8,33%); ambiente físico da escola (três ações; 8,33%); e serviços de saúde na escola (três ações; 8,33%).

## DISCUSSÃO

Esta pesquisa documental apresentou um mapeamento de ações que pode ser uma ferramenta de subsídio para o planejamento e implementação de estudos e programas de intervenção que almejam desenvolver a promoção da atividade física e saúde nas escolas no contexto brasileiro e que estejam alicerçadas pelas recomendações da EPS-OMS.

O mapa de estratégias tem uma maior quantidade de ações voltadas à governança. Em particular, as ações sobre políticas e recursos governamentais e da escola são direcionadas a uma governança que envolve a disponibilidade de recursos financeiros e materiais a serem destinados às escolas para possibilitar uma rede educacional que esteja comprometida com a promoção da saúde. Isso pontua que a agenda da promoção da atividade física e saúde nas escolas passa pela garantia de recursos, governabilidade e compromisso político de construir ambientes com condições e oportunidades favoráveis para a atividade física na vida dos escolares e da comunidade escolar^(2-18)^.

Sete ações foram mapeadas sobre a liderança e gestão escolar, merecendo destaque por incluir a comunidade nas ações de promoção da atividade física e saúde na escola. Isto é um desafio, uma vez que alternativas terão de ser pensadas e trabalhadas para envolver os indivíduos de forma contínua. Para que as ações de promoção da saúde tenham melhor valor agregado na vida dos participantes, elas precisam se manter por tempo prolongado. Essas ações possuem múltiplas influências na vida das pessoas desse processo, pois, para perdurar as condições saudáveis, precisa-se de uma rede de apoio que coincida com essas perspectivas. Ou seja, a construção de uma proposta EPS envolve aspectos contínuos da promoção da saúde^(30,31)^.

As 16 ações para estruturação do contexto escolar tiveram uma maior concentração de ações que envolvem os componentes curriculares (n=7). Isso denota parte dos desafios encontrados na proposta, dado que centraliza no currículo responsabilidades relacionadas a propósitos diferentes, como o desempenho acadêmico. Estudo aponta que diversos fatores contribuem para o desempenho acadêmico, com destaque para o currículo em ambientes com aprendizagem colaborativa intersetorial^(32)^.

A maioria das ações observadas envolvia o desenvolvimento das atividades físicas nas escolas, abordando temas com dimensões de práticas saudáveis com enfoque tanto a nível individual quanto a nível coletivo^(12,30)^. Isso vai de encontro ao modelo EPS, uma vez que as dimensões devem envolver práticas de promoção de saúde que vão além da atividade física, incluindo uma perspectiva integral e uma visão holística do desenvolvimento humano. Ou seja, para além de um aspecto comportamental, é necessário considerar os aspectos sociais, culturais e emocionais que influenciam e precisam ser abordados nas políticas de promoção da saúde escolar^(9,33)^. Nesse sentido, podemos dizer que a promoção da atividade física no ambiente escolar tende a ganhar um enfoque ampliado quando é tratada como uma questão pedagógica vinculada à educação para a saúde^(34,35)^.

As ações ainda surgem, na maioria das vezes, de planos nacionais de atividade física^(12,13,17,31)^. Diante disso, muitas centram-se na realização de atividade física no ambiente escolar, sendo observado como umas das estratégias prioritárias nos documentos. Reforça-se a importância desse elemento na promoção da saúde escolar, mas chama-se a atenção para a importância de se ampliar ações que envolvam aspectos relacionados à saúde mental dos integrantes da escola. Estudos apontam para a necessidade de se abordar essa temática, já que repercute diretamente na saúde física dos estudantes^(36-39)^.

Tem-se a necessidade de alterações no currículo. Contudo, essa estratégia possui entraves de um sistema em que as diretrizes nacionais se pautam em divisões de cunho biologista, esquecendo a intersetorialidade das temáticas. Por isso, os currículos reproduzem vieses tecnicistas e a estratégia EPS pressupõe práticas de prevenção e promoção interrelacionadas.

### Limitações do estudo

Algumas limitações deste estudo precisam ser destacadas. A busca foi restrita a documentos publicados em inglês e português. Isso deve-se também por causa do modelo EPS ainda não ser difundido de forma ampla, sendo os estudos encontrados provenientes de ações mais abrangentes de saúde na escola e com temáticas específicas como citado anteriormente. Ainda, a convergência das 36 ações representa uma estratégia para dimensionalizar várias ações. Contudo, detalhes de cada possibilidade de ação acabam ocultados ao agrupá-los. Portanto, a análise da convergência das 36 ações pelos leitores deve ser acompanhada através da lista inicial das ações (ver material suplementar 1), para que seja possível analisar e adequar os detalhes das ações a cada realidade e possibilidade.

### Contribuições para as áreas da enfermagem, saúde, ou políticas públicas

Os documentos com padrões e indicadores da EPS-OMS^(2,19)^ direcionam suas recomendações para os grupos de atores da escola que podem colaborar para a promoção da saúde no referido contexto, incluindo pesquisadores sobre saúde na escola, formuladores de políticas e programas, gestores escolares, representantes de conselhos e comitês escolares, professores, profissionais de saúde, estudantes, e familiares. Para o contexto brasileiro, espera-se que o mapeamento de ações aqui apresentado possa apoiar esses atores em suas ações, com potenciais implicações para as práticas pedagógicas e de pesquisa, a saber:

- As ações de governança (padrões 1 a 4) podem ser consideradas por gestores e profissionais de saúde (em diálogo com os gestores e profissionais da educação) para alinhar políticas públicas e programas municipais, estaduais e federais na elaboração e gestão de programas de saúde na escola. As redes de ensino podem incluir em seus planos de execuções de políticas de saúde pública o incremento de ações baseadas neste mapa de estratégias adaptadas às diferentes realidades e que nutram um sistema com maior potencial de implementação, efetividade e sustentabilidade;
- Os gestores, coordenadores e professores das escolas também podem englobar a implementação das ações da EPS-OMS na rotina escolar, incluindo no Projeto Político Pedagógico para organizar a política, os recursos, as metodologias, e o envolvimento e engajamento social na promoção da atividade física e saúde na escola.
- Professores tanto de componentes curriculares convencionais (educação física, matemática, ciências, artes, entre outros) quanto que atuam em escolas com propostas curriculares inovadoras (com componentes curriculares eletivos ou de base diversificada) podem considerar as ações curriculares aqui propostas para a formulação de projetos e ações pedagógicas como forma de garantir uma abordagem intersetorial e interdisciplinar para a aprendizagem em saúde que tenha significado real na vida das pessoas;
- Toda a comunidade escolar pode apoiar a implementação de ações para o desenvolvimento do ambiente físico e socioemocional que favorecem o cuidado em saúde e que sejam adequadas às diferentes realidades e contextos. Nesse sentido, a comunidade deve ser estimulada pela gestão participativa no desenvolvimento de uma política coletiva.

Este estudo proporciona a base que alcançará a criação de propostas com refinamento científico de ações e estratégias, as quais incluem os atores sociais envolvidos nesse processo. Pesquisas futuras podem incluir a implementação dessas ações e estratégias em diferentes contextos locais e regionais de seu país, visto que proporcionará uma avaliação mais abrangente. Nesse sentido, uma avaliação da implementação de ações e estratégias desta natureza é relevante, considerando aspectos econômicos, sociais e culturais dos países.

## CONCLUSÕES

O mapeamento das ações baseadas no modelo EPS-OMS permitiu identificar a convergência de 36 ações que podem servir de base para o desenvolvimento da governança e organização de escolas brasileiras, favorecendo a promoção da atividade física e saúde à luz dos modelos e padrões internacionais de promoção da saúde na escola. Estudos e intervenções podem considerar e se apoiar nesse mapeamento para impulsionar a implementação de políticas públicas e programas de educação para a saúde e promoção da saúde no Brasil e outros contextos similares.

## Data Availability

Os dados de pesquisa estão disponíveis em repositório: https://doi.org/10.48331/scielodata.8WDPXC.
